# Towards Onsite Age Estimation of Semen Stains Using Fluorescence Spectroscopy

**DOI:** 10.3390/s23136148

**Published:** 2023-07-04

**Authors:** Nihad Achetib, Caren C. Leemberg, Mathijs M. P. Geurts, Paul R. Bloemen, Richard M. van den Elzen, Maurice C. G. Aalders, Annemieke van Dam

**Affiliations:** 1 Department of Biomedical Engineering and Physics, Amsterdam University Medical Centers (UMC), Location AMC, University of Amsterdam, Meibergdreef 9, 1105 AZ Amsterdam, The Netherlands; n.achetib@amsterdamumc.nl (N.A.); p.r.bloemen@amsterdamumc.nl (P.R.B.); r.m.vandenelzen@amsterdamumc.nl (R.M.v.d.E.); m.c.aalders@amsterdamumc.nl (M.C.G.A.); 2 Methodology Research Program, Amsterdam Public Health Research Institute, Amsterdam University Medical Centers (UMC), Location AMC, University of Amsterdam, Meibergdreef 9, 1105 AZ Amsterdam, The Netherlands; 3 Co van Ledden Hulsebosch Center (CLHC), University of Amsterdam, Science Park 904, 1098 XH Amsterdam, The Netherlands; 4 Department of Forensic Science, Amsterdam University of Applied Science, Tafelbergweg 51, 1105 BD Amsterdam, The Netherlands

**Keywords:** time of deposition, forensics, fluorescence spectroscopy, semen

## Abstract

The age estimation of biological traces is one of the holy grails in forensic investigations. We developed a method for the age estimation of semen stains using fluorescence spectroscopy in conjunction with a stoichiometric ageing model. The model describes the degradation and generation rate of proteins and fluorescent oxidation products (FOX) over time. The previously used fluorimeter is a large benchtop device and requires system optimization for forensic applications. In situ applications have the advantage that measurements can be performed directly at the crime scene, without additional sampling or storage steps. Therefore, a portable fiber-based fluorimeter was developed, consisting of two optimized light-emitting diodes (LEDs) and two spectrometers to allow the fluorescence protein and FOX measurements. The handheld fiber can be used without touching the traces, avoiding the destruction or contamination of the trace. In this study, we have measured the ageing kinetics of semen stains over time using both our portable fluorimeter and a laboratory benchtop fluorimeter and compared their accuracies for the age estimation of semen stains. Successful age estimation was possible up to 11 days, with a mean absolute error of 1.0 days and 0.9 days for the portable and the benchtop fluorimeters, respectively. These results demonstrate the potential of using the portable fluorimeter for in situ applications.

## 1. Introduction

Convicting perpetrators of sexual assault is a challenge due to lack of evidence and eyewitnesses, resulting in a low conviction rate [1]. Factors such as the victim’s use of alcohol or drugs, the absence of physical injuries, and the credibility of the victim can weaken their case, leaving it to be a matter of the suspect’s word against theirs [2,3]. Furthermore, when the assailant has a prior or ongoing sexual relationship with the victim, DNA evidence is of limited value unless the suspect denies sexual intercourse [4]. Knowledge about the age of the semen traces could provide essential information that can be used in reconstructing the timeline of events [5]. Furthermore, knowing the time of deposition can help to decide which samples should be further investigated. Thus, an age-estimation method could improve the quality of crime-scene processing and workflow by reducing the time and resources spent on irrelevant biological traces and strengthening the prosecution’s case in court. However, developing an age-estimation method is difficult due to large inter-and intradonor variation in the chemical composition of the biological traces. Only a few studies have been performed to develop a method to estimate the age of semen stains. These studies investigated degradation markers including semen-specific enzymes and RNA [6,7]. None of these approaches resulted in a robust method until recently [8]. Achetib et al. adapted a method based on fluorescence spectroscopy that is able to estimate the time of deposition of fingermarks for the use of semen stains [9]. This method utilizes the natural fluorescence of biological stains to measure key elements of the ageing process, including the degradation rate of proteins and the formation of fluorescent oxidation products (FOX) [10,11]. These reactions can occur when biological stains are exposed to air, including the oxidation of unsaturated lipids and the formation of reactive oxidation product (LipOX) [9,12] To estimate the age of semen stains we use the intrinsic fluorescence to predict the concentration of the two fluorophore groups. The method does not determine the concentration of protein and FOX directly, but uses the total autofluorescence intensities of the two recorded fluorescence spectra of the two fluorophore groups. Thereby assuming that the intrinsic fluorescence is proportional to the amounts of protein and FOX.As LipOX and FOX are complex mixtures of known and unknown oxidation products, the stoichiometric constants were set to 1 (LipOX + Protein → FOX, (2)). The protein fluorescence will not always decay to zero, as such, the protein fluorescence was divided into two parts. One part is assumed to interact with LipOX, while the other fraction does not. Since LipOX is generated by the oxidation of unsaturated lipids and consumed by the oxidation of proteins, we assume LipOX to remain constant. Thus, the ageing process is modeled using a first-order rate equation that describes the stoichiometry of the protein and FOX behavior over time, resulting in an ageing function. For the derivation of the ageing function, we refer to the supporting information of the study of van Dam and Schwarz et al. Using the model, the age estimation of semen stains was possible up to 16 days, with a mean absolute error less than two days [9]. The method was found to be robust and has the potential to be implemented in forensic casework because of its nondestructive, rapid, and quantitative features. However, one practical limitation is the size and weight of the large benchtop fluorimeter, which hampers the applicability of this method directly at the crime scene. The portability of our method would improve the quality of the crime-scene processing as more information could be extracted in a shorter time span and additional sampling or storage steps would not be needed. Therefore, the aim of our study was to develop a portable fluorimeter that enables fast, onsite, and noninvasive age estimation of semen stains with (at least) similar accuracy compared to the benchtop fluorimeter. The ageing kinetics of semen stains were measured with both fluorimeters, and we benchmark the portable fluorimeter against the benchtop fluorimeter. To this end, two sets of semen stains were created, of which the first set was used for the optimization of our model with 30 semen stains obtained from ten donors. The second set, which consisted of 15 semen stains from five donors, was used to validate our approach. The performance of the portable and benchtop fluorimeters in a lab setting was evaluated at time intervals ranging from 0 to 27 days.

## 2. Materials and Methods

### 2.1. Sample Collection and Preparation

This study was conducted according to the Netherlands Code of Conduct for Research Integrity and the research code of the Amsterdam UMC, Academic Medical Center. Informed written consents were obtained from all semen donors by the fertility clinic of the Amsterdam UMC. Semen samples (without anticoagulants) were obtained from fifteen anonymous donors and stored at −80 °C until use. These samples had been collected between 2016 and 2021 from the fertility clinics at the Amsterdam Medical Center and the Isala Hospital. Before use, samples were thawed and vortexed to obtain a homogenous solution. Three semen stains per donor with a volume of two μL each were deposited on a TLC plate (Silicagel 60, Merck KGaA, Darmstadt, Germany). A total of 45 (*n* = 15) semen stains was ultimately prepared, encompassing an optimalization set of 30 (*n* = 10) semen stains and a test set of 15 (*n* = 5) semen stains. Measurements were started at two different dates to acquire data with continuous days, i.e., without a weekend gap. These samples were measured on 20 different time points, from 0 to 27 days.

### 2.2. Instruments

**Figure 1:**
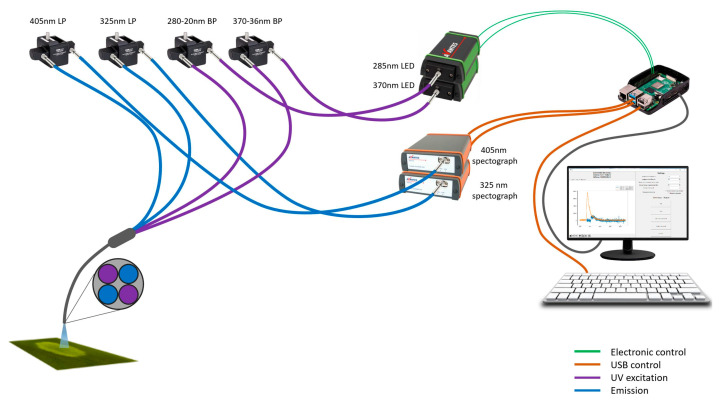
A graphical illustration of the developed portable fluorimeter.

The fluorescence measurements were performed with the commercially available LS55 Luminescence spectrometer equipped with a fiber optic accessory (Perkin Elmer, Waltham, MA, USA). Meanwhile, the portable fluorimeter was in-house built within the Biomedical Engineering and Physics Departments of the Amsterdam UMC, University of Amsterdam (Figure 1). The portable fluorimeter equipped with a handheld fiber consists of two light-emitting diodes (LEDs) emitting at 285 and 370 nm (Avalight-HPLED, Avantes, Apeldoorn, The Netherlands) and two bandpass filters at 280 nm and 370 nm, with a 20 nm and 36 nm bandpass, respectively. To measure the protein and FOX emissions, the portable fluorimeter consists of two fiber spectrographs (Avantes AvaSpec-ULS2048CL-EVO and AvaSpec-ULS2048-USB2-VA-50, Avantes, Apeldoorn, The Netherlands) with longpass filters to record emissions between 325 and 1100 nm and 405 and 1100 nm, respectively. Furthermore, to control the LEDs and spectrographs for data collection, a Raspberry Pi 3B with a mouse, keyboard, and monitor was used in combination with custom acquisition software written in Python.

### 2.3. Spectra Collection and Data Preprocessing

Fluorescence emission spectra were recorded using the portable fluorimeter and the benchtop fluorimeter for each semen spot. For the portable fluorimeter, the emission wavelengths were recorded at the ranges of 325–1100 nm (excitation at 285 nm) and 405–1100 nm (excitation 370 nm) with an integration time of 4 s and 1 s, respectively. For the benchtop fluorimeter, the emission wavelengths were recorded at the wavelength intervals of 325–500 nm (excitation at 285 nm) and 400–500 nm (excitation at 370 nm) with excitation and emission bandwidths of 10 nm and 7.5 nm, respectively. For the portable fluorimeter, from each sample, two measurements were required: the dark response of the system and the response of the sample upon excitation. The dark measurement was subtracted from the sample fluorescence. This correction is already incorporated in the benchtop fluorimeter. All measurements with both fluorimeters included additional background-correction by subtracting the intrinsic fluorescence of the TLC plate. The area under the curve (AUC) was calculated for FOX (FOX_fl_: excitation of 370 nm), while for the AUC of protein (Protein_fl_: excitation of 285 nm), an additional step was needed to correct for the presence of nonprotein fluorophores or contaminants from the substrate. As such, a reference spectrum was constructed by averaging the measured spectra of thirteen fresh semen samples.

On a silica-coated TLC plate, which was prewashed with methanol (Sigma-Aldrich, Saint Louis, MO, USA) and activated for 30 min at 120 °C, 2 μL of semen sample was deposited in duplicate. The TLC plate was developed with chloroform/methanol (30 mL/120 mL, Merck KGaA, Darmstadt, Germany) solution as mobile phase to elute all nonprotein-related fluorophores and contaminations. The dried semen residues were measured using both fluorimeters (excitation: 285 nm). The average of the measured semen residue spectra yielded solely protein fluorescence which was fitted to each sample fluorescence spectrum using the weighted least-squares method [9]. The AUC of the fitted spectrum was calculated to estimate the protein fluorescence (Protein_fl_).

### 2.4. Age Estimation

The age estimation of semen stains was performed using the method described by Achetib et al. [9]. The protein- and FOX-specific fluorescence spectra were recorded at consecutive time points, and subsequently, the area under the curve (AUC) was computed (Protein_fl_ and FOX_fl_). The ratio of the area under the curve for protein and FOX was calculated for each time point to create time series of AUC ratios. In order to estimate the time of deposition of the semen stains, the ageing model was fitted to these time series of AUC ratios [9,11]. The mathematical equation models the behavior of the protein and FOX over time [Equation (1)]:

(1) $$f\left(t\right)=\frac{{\left[\mathrm{Protein}\right]}_{t}}{{\left[\mathrm{FOX}\right]}_{t}}=\frac{\left(f_{0}-f_{\infty }\right)e^{-kt}+f_{\infty }\left(f_{0}+1\right)}{-\left(f_{0}-f_{\infty }\right)e^{-kt}+f_{0}+1}$$

For age estimation, the first measurement was conducted at an unknown time after deposition of the semen stain, referred to as *t*_0_ days. Subsequently, measurements were performed on several days after *t*_0_, thereby generating multiple values for *f*(*t*_0_ + *t*_measured_), where *t*_measured_ is the time since the first measurement. The equation was solved by fitting the ageing function [Equation (1)] to the data points using a nonlinear least-squares method to estimate the parameters *f*_0_, *k*, and *f*∞. The parameter *f*_0_, which is the value of *f*(*t*) at *t* = 0, was empirically determined, due to the high variability between donors as has been described by Achetib et al. in previous work [9]. To account for interdonor variability, four empirically obtained *f*_0_ values were used for all donors. Different *f*_0_ values were applied for both devices, due to their different technical specifications and the detection mechanisms of the devices. These *f*_0_ values corresponded to donors with high, medium, and low Protein_fl_/FOX_fl_ ratios. Specifically, for the portable fluorimeter, the *f*_0_ values were 2 (low), 5, 8 (medium), and 11 (high), while for the benchtop device, the *f*_0_ values were 6 (low), 9, 12 (medium), and 15 (high). By averaging the estimates obtained using these four *f*_0_ values individually at each time point, the age of semen traces could be calculated. The *f*∞ was determined by averaging the last three data points of the time series of each sample when no dynamics in ageing kinetics could be observed. The third unknown, which is the rate constant (*k*), was estimated with a linear fit applied to the first five data points of each time series. The function was fitted to the time series (ranging from 0 to 27 days) for each semen stain. Subsequently, the first time point was removed from the recorded measurement series, and the ageing function was fitted. The procedure was repeated for every recorded measurement to simulate increasing ages for *t*_0_. To ensure reliable age estimations, the data and the fitting of the ageing function had to pass our inclusion and exclusion criteria.

#### Exclusion Criteria

Using the test set, the fit of the ageing function was considered reliable if the following criteria were met:

(a) R2 of the fit of the ageing function exceeded 0.85;
(b) Six or more were nonconstant data points;
(c) The difference between the Protein_fl_/FOX_fl_ ratio of the first measurement and *f*_0_ was at least 0.10;
(d) The signal-to-noise ratio, defined as the extent of decay of the ageing curve relative to the root mean square error of the fit, exceeded 3.92.

The settings of our criteria and the *f*_0_ values were determined using the training set and were subsequently applied on the test set.

MATLAB^®^ (The Mathworks Inc., Natick, MA, USA) custom-written scripts were used for all data analysis.

## 3. Results

### 3.1. Protein and FOX Emission Spectra

**Figure 2:**
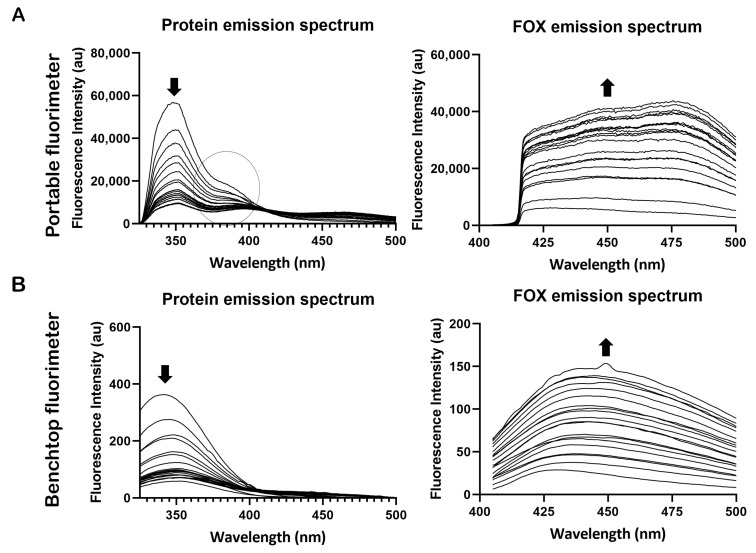
Typical example of the protein and fluorescent oxidation products (FOX) spectra of a semen stain over time: (**A**) semen stains measured with the portable fluorimeter show two peaks in the protein spectra, in the wavelength ranges of 325–375 nm (circle) and 375–425 nm; (**B**) semen stains measured with the benchtop fluorimeter show one clear peak in the protein spectra, in the wavelength range of 325–375. For FOX fluorescence, no clear peaks could be distinguished with both fluorimeters. Please note that protein fluorescence decreased over time, while FOX fluorescence increased (indicated by black arrows).

With both fluorimeters, the decrease in protein fluorescence and the increase in FOX fluorescence over time could be monitored. However, spectral differences were observed between the emission spectra of the two fluorimeters. With the portable fluorimeter, two peaks can be distinguished in the protein spectrum in the wavelength ranges of 325–375 nm and 375–425 nm, see Figure 2A. The peak between 375 and 425 nm was not observed when measuring with the benchtop fluorimeter (Figure 2B). To allow comparison between the two fluorimeters, only the peak in the wavelength range of 325–375 was used. For the FOX spectrum, the spectral shape also differs when measured with both fluorimeters; however, no clear peaks could be distinguished, see Figure 2.

### 3.2. Ageing Kinetics

**Figure 3:**
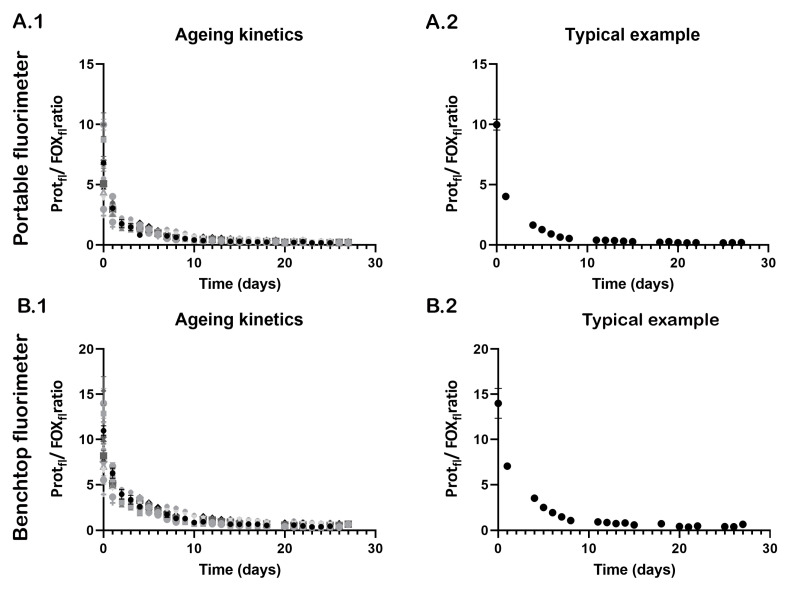
Ageing kinetics of semen stains over time measured with the portable (**A.1**,**A.2**) and benchtop (**B.1**,**B.2**) fluorimeters: ageing curves of fifteen donors (**A.1**,**B.1**), whereby each symbol (shape and colors) represents the average Protein_fl_/FOX_fl_ of triplicate semen stains from one donor and the error bars represent the standard deviations. A typical example of the mean Protein_fl_/FOX_fl_ and standard deviations of three semen stains from the same donor (donor H) are shown for the portable (**A.2**) and benchtop (**B.2**) fluorimeters. Please note that the intravariability between the ratios of the three semen stains is low and mostly occurs at younger ages.

The ageing kinetics of semen stains measured with the portable and benchtop fluorimeters are presented in Figure 3. Similar decay kinetics were observed, despite the differences in their fluorescence spectra. Over time, the Protein_fl_/FOX_fl_ ratios decreases until no dynamics in the reaction kinetics can be observed. Note that little variation was observed within the triplicate measurements (Figure 3A.2,B.2).

**Figure 4:**
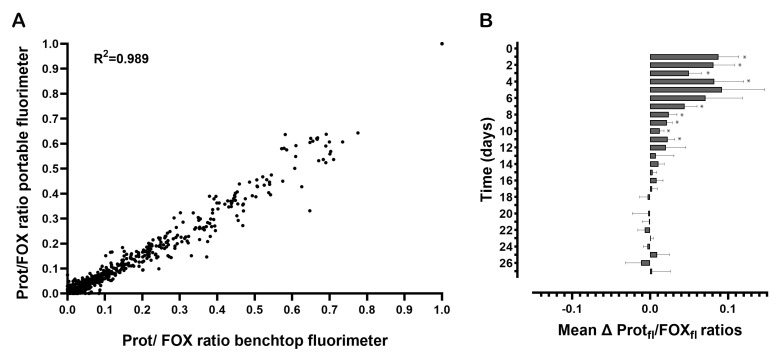
Protein_fl_/FOX_fl_ ratios of each sample at different ages measured with the portable and benchtop fluorimeters: (**A**) the correlation of the normalized Protein_fl_/FOX_fl_ ratios between both fluorimeters; (**B**) the average difference in mean Protein_fl_/FOX_fl_ ratios and the upper and lower bounds of the 95% confidence interval between the two fluorimeters per time point. Here, the positive Protein_fl_/FOX_fl_ ratios show how much higher the ratios measured by the benchtop fluorimeter were than those measured by the portable device. * difference significant at *p* < 0.001.

To allow the comparison of Protein_fl_/FOX_fl_ ratios over time, these were normalized by scaling the lowest ratio to 0 and the highest to 1 with each time series. The normalized Protein_fl_/FOX_fl_ ratios of each sample measured by the portable fluorimeter were plotted against the normalized Protein_fl_/FOX_fl_ ratios that were measured with the benchtop fluorimeter, see Figure 4. A Pearson rho test showed a strong correlation between the Protein_fl_/FOX_fl_ ratios measured with the portable and benchtop fluorimeters (Pearson correlation coefficient = 0.99, *p* = 0.000). The average differences of the mean Protein_fl_/FOX_fl_ ratios between the two fluorimeters per time point are shown in Figure 4B. The Protein_fl_/FOX_fl_ ratios of the benchtop fluorimeter were at a maximum (*t* = 5 days) 9% higher than those measured by the portable fluorimeter.

### 3.3. Age Estimation of Semen Stains

**Figure 5:**
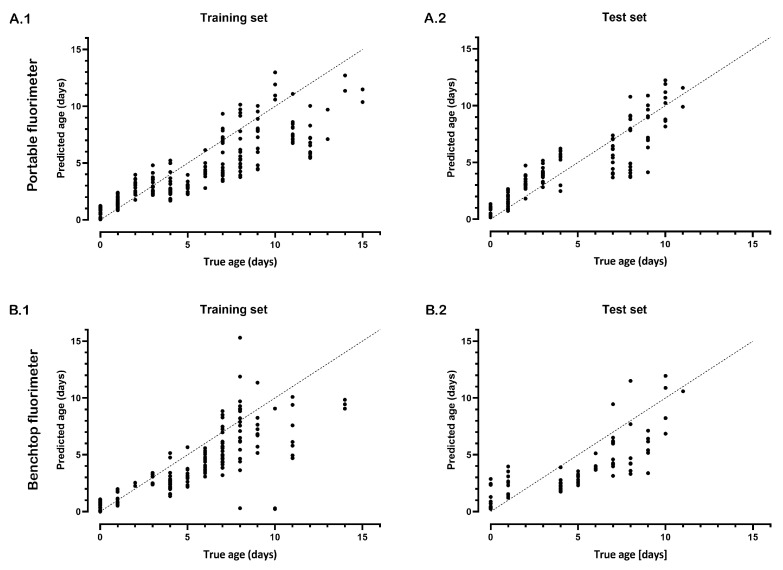
Age estimation of semen stains measured with the portable and benchtop fluorimeters: age estimation with the portable (**A.1**,**A.2**) and benchtop (**B.1**,**B.2**) fluorimeters using a training (**A.1**,**B.1**) and test set (**A.2**,**B.2**). The dots represent the average age estimation of the four different *ƒ*_0_ values of each semen stain for each time point.

First, the model was optimized using a training set and four different *f*_0_ values as described earlier. The ageing function was fitted to the time series of the Protein_fl_/FOX_fl_ ratios using these *f*_0_ values, and the predicted ages were averaged for each time point. A strong correlation was observed between the true and estimated age of the semen stains of the optimalization set measured with the portable (Spearman rho = 0.91, *p* = <0.001) and benchtop fluorimeters (Spearman rho = 0.87, *p* = <0.001), see Figure 5. Similar to the optimalization set, the test set showed a very strong correlation between the true and estimated age of the semen stains using the portable (Spearman rho = 0.94, *p* = <0.001) and bench top fluorimeters (Spearman rho = 0.95, *p* = <0.001). For the optimalization sets with both fluorimeters, age estimation was possible up to 15 days with a mean absolute error (MAE) of 1.9 days. For the test sets (using the optimized criteria determined with the optimalization set), age estimation was possible up to 11 days with a MAE of 1.0 days and 0.9 days for the portable fluorimeter and benchtop fluorimeter, respectively.

## 4. Discussion

In this work, the predictive ability of an in-house-built portable fluorimeter was compared to a commercially available benchtop fluorimeter. Previous work on the age estimation of semen stains was performed using the benchtop fluorimeter [9]. Although promising results have been demonstrated, for implementation in forensic casework, it is highly important to enable onsite analysis. Here, we developed an in-house-built portable fluorimeter that could easily be transported to a crime scene.

With both fluorimeters, the ageing kinetics of semen stains could be measured; however, spectral differences were observed in the spectra of protein and FOX measurements. The differences in the shape and intensities of the emission spectra were attributed to the differences in the properties of both light sources. The portable fluorimeter uses LEDs for the excitation of semen stains, while the benchtop fluorimeter contains a Xenon lamp as its light source. The power of the LEDs for the protein measurements is 2.3 times higher than the power of the Xenon lamp, which could explain the extra peak at 375–425 nm. The higher excitation intensities from the LEDs could reveal low-abundance fluorophores, which cannot be measured with the Xenon lamp. However, these differences seem to have little effect on our ageing model since a very strong correlation was observed between the ratios of both fluorimeters, and the age estimation was performed with similar accuracies (MAE of 1.0 days and 0.9 days for the portable fluorimeter and the benchtop fluorimeter, respectively). Finally, age estimation was possible up to 15 days with the used study setup; since our model requires at least eleven data points (which are measurements of eleven days in this study), this means that samples of sixteen days or older could not be dated. In this study, we have performed daily measurements, but to allow fast analysis directly at the crime scene, future research should investigate frequent measurements in a shorter time range. If sufficient dynamics can be captured, the existing crime-scene workflow will be minimally affected. In addition, further improvement in the accuracy of our age estimation may be needed, as the true age is underestimated. However, important to note is that there is no method for age estimation of semen stains with such minimal user input and low errors available in the field yet. With the developed portable fluorimeter, we are a step closer to fulfilling the current need in the forensic community for an age-estimation method that can be brought to the crime scene [13]. For forensic investigations, our method could lower the cost by reducing the time spent on irrelevant traces and improve efficiency at the crime scene. While in court, the age of semen stains can be used to determine the weight of the evidence and link the evidence to the time interval in which the crime occurred, which strengthens the prosecution’s case. The method provides high accuracy without destroying the sample. However, more studies are needed to allow the implementation of this method in forensic casework. Future studies will focus on investigating how substrate characteristics and environmental factors will affect the formation of degradation products in semen stains. Different substrates interact with semen in various ways. For porous substrates, such as fabric or paper, various components in semen may diffuse into the substrate pores, which may affect the degradation rates. While with nonporous substrates, the components sit on the substrate and are therefore more exposed to environmental factors. Moreover, the surface chemistry of the substrate can influence the physical and chemical properties of semen. In our previous work, we conducted preliminary experiments that demonstrated the versatility and robustness of our fluorescent-based method for analyzing semen on various forensic-relevant surfaces [9]. In the study, the results showed that the fluorescent protein–lipid oxidation signatures observed on tissue, white tile, and blue and black plastic were similar to those that were observed on the TLC plates [9]. Thus, we expect our method to be applicable to a range of various substrates. Light, temperature, and humidity could also influence the ageing process and require corrections to ensure accurate age estimation under different conditions [14,15,16,17]. The study by van Dam et al. showed that environment, particularly light and temperature, can significantly influence the decay kinetics of fingermarks [11]. Both light and higher temperatures can speed up the degradation process in fingermarks [11]. Since fingermarks were approached as mixtures of protein and lipids, similar effects are expected for other biological samples. However, more studies are needed to determine the potential effects of the environment on decay kinetics.

In previous work, fresh semen stains were used instead of frozen semen stains. From the literature, it is known that freezing could affect protein and lipid oxidation; therefore, the effects of freezing on the ageing process should further be investigated [18]. Furthermore, field validation studies are necessary to evaluate the integration of our portable fluorimeter for the age estimation of biological fluids within the existing crime-scene workflow. In addition, miniaturizing the system and simplifying the use of our custom-written software is needed as the software requires specialized knowledge. This will provide the necessary validation for implementing our method in the forensic field. Finally, the portable fluorimeter could potentially have a broad range of future applicability, including the identification of body fluids using specific fluorescent signatures, the differentiation of human remains based on their luminescence, or monitoring the quality of food [19,20,21,22]. Taken together, these study results have demonstrated the great potential of our portable fluorimeter for forensic casework with accuracies that are equal to benchtop instrumentation.

## 5. Conclusions

In the present study, we have demonstrated the applicability of our noninvasive, low-cost, fluorescence-based age-estimation method for semen stains. The performed lab validations in this study have demonstrated that age estimation with the portable fluorimeter can be performed with similar accuracies as a benchtop fluorimeter. There is currently no method available with a high level of accuracy for the age estimation of semen stains. Our method has the potential to enhance the quality of crime-scene processing and meets the forensic community’s demand for the quicker acquisition of information without disrupting the existing crime-scene workflow and risking contamination.

## Data Availability

The datasets generated and analyzed in the current study are available from the corresponding author on reasonable request.
